# Enhancing veterinary systematic and scoping reviews reporting for better identification and dissemination in VetSRev

**DOI:** 10.18849/ve.v10i4.721

**Published:** 2025-10-09

**Authors:** Elisa Martello+, Lisa Morrow+, Heather Moberly+, Douglas Grindlay+, Marnie Brennan+

**Keywords:** EVIDENCE SYNTHESIS, PRISMA, SCOPING REVIEW, SYSTEMATIC REVIEW, VETSREV

## Abstract

Veterinary medicine advances rapidly, generating extensive amounts of research. Systematic and scoping reviews synthesise evidence to help guide clinical decisions, improve welfare, influence policy, and shape research priorities. Unlike narrative reviews, they employ transparent, rigorous methods. This commentary presents insights from curating VetSRev, a more than a decade-old, freely accessible repository containing an interactive list of published veterinary systematic and scoping reviews. Accessible, high-quality reviews strengthen public and owner trust, improve animal welfare, and promote evidence-based practice. Following established protocols and guidelines ensures quality, visibility, and VetSRev inclusion, thereby disseminating the reviews to users, advancing knowledge, informing research priorities, and enhancing clinical decision-making in veterinary medicine.

## Introduction

The field of veterinary medicine is constantly evolving, with new diagnostic and therapeutic approaches being developed every day, including investigations of new ways to use existing tools and skills. As this continues, practitioners and researchers face an ever-increasing volume of evidence in the published literature. Well-conducted systematic reviews and scoping reviews play a crucial role in informing evidence-based practice. Systematic reviews are comprehensive and structured analyses of existing research studies to answer a specific research question, involving rigorous methods to identify, evaluate, and synthesise relevant literature (Sargeant & O’Connor, 2020). On the other hand, scoping reviews give an overview of existing literature to identify the extent, range, and nature of available research on a particular topic, often used to clarify the research focus before planning new projects or systematic reviews (Sargeant & O’Connor, 2020). These reviews offer a comprehensive and reliable way to synthesise and appraise a vast body of knowledge, to inform clinical decision-making, enhance animal welfare, and guide future research. These reviews are different to the more traditional narrative reviews, which usually lack any description of the methodology used to identify the relevant papers included. This makes it difficult for readers to understand why some papers have been highlighted and why others have been omitted, potentially leading to a biased selection of papers.

The purpose of this commentary is to signpost the most appropriate ways to conduct and report systematic reviews and scoping reviews, highlighting issues from our perspective as curators of VetSRev for more than ten years (since 2013). VetSRev is a freely accessible online repository (VetSRev database, 2021) specifically designed to contain an interactive list of the systematic reviews and scoping reviews published to date that are relevant to veterinary medicine (Grindlay et al., 2021). The published literature pertaining to veterinary medicine is not indexed in a single database, which makes locating existing systematic reviews and scoping reviews challenging (Grindlay et al., 2012), hence the need for a specific repository. VetSRev has served as a valuable resource for researchers and practitioners in the veterinary community for the past decade. We are hoping that the recommendations made in this commentary, which are based on our experiences curating VetSRev, will help authors conduct and report their reviews robustly, facilitating our identification of relevant studies for inclusion in VetSRev.

## General considerations on the importance of well-conducted systematic reviews and scoping reviews in veterinary medicine

Systematic reviews and scoping reviews play a crucial role in informing and guiding scientists, policymakers, public health professionals, clinicians, and the public. They employ rigorous methodologies to find all relevant available studies.

Before beginning the process of conducting reviews, it is recommended that authors plan the scope and approach for the review they are undertaking, and to register a protocol of this information in recognised public platforms like International Prospective Register of Systematic Reviews (PROSPERO) (National Institute for Health and Care Research, 2023), Generalized Systematic Review Registration Form (OSFHOME, 2023), or Systematic Reviews for Animals & Food (SYREAF) depending on the topic under investigation (Systematic Reviews for Animals & Food, 2023). This practice promotes transparency, helps prevent selective reporting, and reduces bias.

Collaborative efforts involving multiple authors and professionals from various disciplines, including methodological specialists such as epidemiologists, are highly recommended in the review process to ensure a comprehensive and well-conducted review. This is because conducting a comprehensive review requires a diverse range of skills and involves various tasks such as searching, double screening, risk of bias, data extraction or other assessments of the included studies. The involvement of an experienced librarian or information specialist is critical to the success of any review. Searching is a skilled process and information specialists can assist in selecting appropriate keywords, refining search strategies, and identifying relevant databases to search for papers, which can be the difference between finding or not finding relevant studies (Rethlefsen et al., 2021).

To ensure the reliability and usefulness of these reviews, it is imperative that they are both conducted and reported in a meticulous manner and that these distinct processes are not conflated. A poorly conducted review can be well reported, and a methodologically sound review may be poorly reported and hard to appraise. In the absence of detailed methodological guidance for conducting systematic reviews or scoping reviews in the field of veterinary medicine (although articles do exist outlining the process (O'Connor & Sargeant, 2015; Sargeant & O’Connor, 2020)), it is advisable to follow or modify established methods from human medicine such as those published by Cochrane (Cochrane Library, 2023), the JBI (JBI, 2023; Aromataris et al., 2024) and the Campbell Collaboration (Campbell Collaboration, 2023).

For reporting, there are veterinary specific guidelines for other types of research approaches (Winder et al., 2019), but not yet for systematic reviews or scoping reviews. It is advisable to follow the updated  “Preferred Reporting Items for Systematic Reviews and Meta-Analyses” (PRISMA statement) reporting guidelines for systematic reviews (Page et al., 2021) and its extension that cover scoping reviews (Tricco et al., 2018). These methods and reporting guidelines provide frameworks and standardised approaches for both conducting and reporting reviews, ensuring transparency, clarity, methodological rigor, and reliability of the review process. Some of the features within these reporting guidelines are used to identify the reviews eligible to be added to VetSRev, and by adopting these, researchers can facilitate the inclusion of their work in VetSRev. In the following section we underline what researchers can do to make it easier for their reviews to be identified and included in VetSRev.

The responsibility of conducting and submitting a systematic review or scoping review lies with the authors. However, journals have a role to play in improving the quality of reviews that are published by requiring authors to use the appropriate reporting guidelines during writing. In previous research it was found that relatively few veterinary journals recommended the use of reporting guidelines (Grindlay et al., 2014; Bennett, 2022). Following the authors' submission, it is the responsibility of journal editors to recruit reviewers, including information specialists or librarians (Librarian Peer Reviewer Database), who have the appropriate skills to review these manuscripts, and possibly encourage reporting guidelines to be used as a guide whilst reviewing manuscripts. Ideally this assessment would contribute to decisions whether to accept or reject submissions that do not comply (Toews, 2017).

## Lessons learned from curating a veterinary systematic review and scoping review database (VetSRev)

To find published systematic reviews and scoping reviews, we recommend VetSRev which is produced by the Centre for Evidence-based Veterinary Medicine (CEVM) at the University of Nottingham, UK. This unique repository, which was launched publicly in 2013, offers a comprehensive collection of systematic reviews and scoping reviews, thus providing a high-quality evidence pool for veterinary medicine and science.

VetSRev is overseen by a team of experienced researchers who meticulously evaluate published systematic reviews and scoping reviews identified through comprehensive searches conducted in the PubMed and CAB Abstracts databases. These searches utilise specialised search filters to identify relevant SR and scoping reviews.

The selection process follows a well-defined set of criteria for papers to be included in VetSRev (Grindlay et al., 2021):

- The paper should pertain to topics of interest within the realm of veterinary medicine and science.
- Should contain clear documentation of bibliographic databases.
- Should specify the search strategy.

This information should be present in the abstract and other sections as described in the appropriate reporting guidelines, including supplementary material.

**Figure 1:**
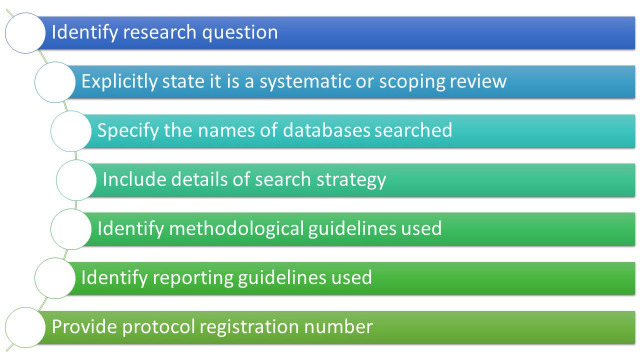
Suggested workflow for undertaking a systematic review or scoping review

There has been previous research looking at the reporting of systematic reviews in veterinary medicine which has identified a general lack of adherence to PRISMA reporting guidelines, generating some useful conclusions for those wanting to report reviews in the future (Sargeant et al., 2021; Tramuta-Drobnis et al., 2025). Our viewpoint here is specifically focused on the information authors should provide in order for us to easily identify and include studies in VetSRev. Leveraging our 10 plus years of experience in collating studies to include in VetSRev, the CEVM team proposes the following suggestions for authors who wish to undertake and publish a systematic review or scoping review. By following these proposed suggestions (Figure 1), authors can facilitate easier and faster categorisation of their work by VetSRev team, allowing for improved dissemination, visibility and accessibility to the veterinary community of researchers and practitioners.

It is crucial to ensure that the title accurately reflects the purpose of the review, providing a concise summary of the research question, and explicitly stating whether the work is a systematic review or a scoping review. This helps set clear expectations from the outset, permitting the easy identification of the type of review being conducted and the topic covered. The abstract should further elaborate on the title by outlining the key components mentioned earlier, providing a brief overview of the research question topic, methodology, and scope of the review. Specifically, to facilitate the screening process and inclusion of published papers in VetSRev, we strongly recommend authors explicitly provide the following information in the abstract: the research question, the names of the databases used for the search, and should specify that a structured search strategy was used. The full text (or methods section) should also include this information as well as full details of the combination of keywords used in the search strategy. However, it is important to note that including the necessary information in the main text and not in the abstract is significantly likely to slow down the selection process carried out by the CEVM team. In addition, although it would seem intuitive, it is essential to ensure that when the methods are reported in the abstract, they must also be thoroughly described and reported in the full text or supplementary material to maintain completeness. We strongly encourage authors to indicate whether they have registered review protocols (ideally mentioning protocol registration numbers) and used established guidance or guidelines for conducting and reporting systematic reviews or scoping reviews such as those mentioned in this article. Providing this information will speed up the screening process for VetSRev.

Ultimately, these measures enhance the completeness and usefulness of the reviews within VetSRev and make this database a comprehensive resource for researchers to use when establishing whether a previous review has already been done, and for practitioners looking to see what evidence is available on their topic of interest.

## Conclusion

Performing and reporting well-conducted systematic reviews and scoping reviews in veterinary medicine in a scientifically rigorous, repeatable, and visible manner offers great advantages for the scientific community and general public. If the required information is reported, the reviews will more easily be included in VetSRev. This will support its aim to be an invaluable resource for clinicians, scientists, public health professionals, and the general public (including animal owners). For the scientific community, these reviews consolidate extensive evidence, providing researchers and clinicians with comprehensive data in the field of veterinary science. For the public, the accessibility of these reviews builds trust in evidence-based practices and equips them with reliable information that directly influences animal welfare and health outcomes. By registering protocols, using defined review methodologies and adhering to established guidelines for systematic reviews and scoping reviews, authors can ensure their reviews meet the necessary standards for visibility and reliability, and we can enhance the dissemination of these works in VetSRev. This will ultimately advance knowledge, enhance clinical decision-making, improve animal welfare, and help to inform research priorities.

## Author contributions

**Elisa Martello**: Conceptualisation, Data Curation, Methodology, Software, Validation, Writing-Original Draft Preparation, Writing-Review and Editing. **Lisa D. Morrow**: Software, Validation, Writing-Review and Editing. **Heather K. Moberly:** Writing-Review and Editing. **Douglas J.C. Grindlay: **Writing-Review and Editing. **Marnie L. Brennan:** Conceptualisation, Methodology, Software, Validation, Resources, Data Curation, Writing-Original Draft Preparation, Writing-Review and Editing, Funding Acquisition.

## ORCiD

Elisa Martello: https://orcid.org/0000-0003-0247-6670

Lisa D. Morrow: https://orcid.org/0000-0003-3001-1088

Heather K. Moberly: https://orcid.org/0000-0002-5080-2656

Douglas J.C. Grindlay: https://orcid.org/0000-0002-0992-7182

Marnie L. Brennan: https://orcid.org/0000-0002-4893-6583

## Conflict of Interest

Elisa Martello was employed by the Centre for Evidence-Based Veterinary Medicine, which owns and operates the VetSRev system. She was not involved in the creation or operation of VetSRev.

## Funding

There was no funding for this article. The initial creation of the database VetSRev, was supported by an unrestricted grant from Novartis Animal Health and the University of Nottingham, UK.
